# Preparation of fluorescent in situ hybridisation probes without the need for optimisation of fragmentation

**DOI:** 10.1016/j.mex.2018.11.015

**Published:** 2018-11-27

**Authors:** Patrick J. McCoy, Anthony J. Costello, Niall M. Corcoran, Christopher M. Hovens, Michael J. Clarkson

**Affiliations:** a Departments of Surgery and Urology, University of Melbourne, Royal Melbourne Hospital, Parkville, Australia; b Australian Prostate Cancer Research Centre Epworth, Victoria, Australia

**Keywords:** Rapid probe construction for Fluorescence in situ hybridization (FISH), Fluorescence in situ hybridization, FISH, Cancer, Translocation, Probe

## Abstract

DNA-fluorescence in situ hybridisation (DNA-FISH) allows visualisation of chromosome organisation and rearrangement. FISH probes are pools of short fluorescently labelled DNA fragments that are often produced from template plasmids that contain large genomic inserts. For effective sample penetration and target hybridisation it is critical that probe fragments are between 200 and 500bp. Production of these short probes requires significant optimisation and can be confounded access to expensive sonication equipment or inherent sequence features that influence enzymatic fragmentation or amplification. Here we demonstrate that effective FISH probes can be prepared without the need for optimisation of fragmentation using a cocktail of two the 4bp recognition sequence restriction enzymes CviQI and AluI.

## Graphical abstract

**Figure:**
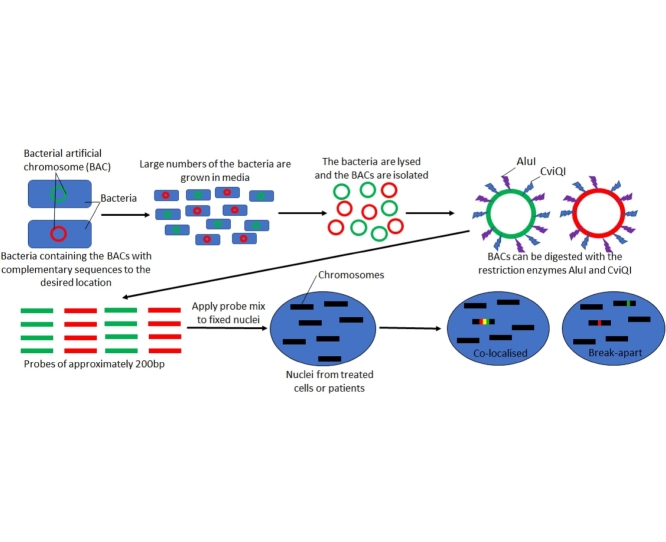

**Specifications table**

**Table:** 

**Subject Area**	
**More specific subject area:**	*Fluorescence* in situ *hybridization (FISH) for the investigation of translocation and fusion genes in cancer*
**Method name:**	*Rapid probe construction for Fluorescence* in situ *hybridization (FISH)*
**Name and reference of original method**	*If applicable, include full bibliographic details of the main reference(s) describing the original method from which the new method was derived.*
**Resource availability**	*If applicable, include links to resources necessary to reproduce the method (e.g. data, software, hardware, reagent)*

## Method details

### Method summary

Production of FISH probes from large genomic insert vectors requires extensive optimisation to produce 200-500bp fragments. Here we demonstrate that suitable fragmentation can be achieved using a cocktail of the restriction enzymes CviQI and AluI.

### Method description

Use of DNA-FISH in clinical cytology labs to identify genome duplications, deletions and rearrangements is routine practice and is an invaluable tool in diagnostic and prognostic analysis of patient samples. Generally probes used in this setting are obtained from commercial sources and are expensive and only available for regions with well-established clinical utility. The advent of next generation sequencing technologies has brought with it knowledge of an increased number of genome rearrangements and motivation to further examine their frequency, distribution and functional significance Morrison et al. [1], Specht et al. [2]. In many cases commercially prepared probes are not available to these regions so they need to be produced in house. The favoured starting material for probe generation are large insert vector libraries containing mapped genomic sequences because they are a readily available source of DNA spanning the region of interest and they can produce probes that hybridise across an extended region to increase signal strength. More often than not, these large insert vectors are maintained in bacteria as low or single copy and are difficult to obtain in any quantity, requiring large volumes of culture and lysis reagents. Once the template has been obtained there are a number of ways of using it to prepare a FISH probe to detect a specific region but they almost all require extensive optimisation to produce a pool of labelled fragments of between 200 and 500bp (Morrison et al. [3],). Here we describe methods for optimised production of bacterial artificial chromosome (BAC) DNA from small culture volumes and a strategy for preparation of FISH probes without requirement for optimisation of fragmentation to produce a pool of 200-500bp labelled fragments. Trials conducted on a number of human BAC clones and genomic DNA indicate that this will work on any large insert vector template.

To obtain microgram amounts of BAC DNA of suitable quality for FISH probe generation we modified the protocol of QIAGEN plasmid mini kit (QIAGEN, Melbourne, Australia) (see supplemental material). This method routinely yields more than 10ug of pure BAC DNA from 50 ml of starter culture. Columns used for preparation are able to be recycled as described in Siddappa et al. [4]. In summary, this method uses an alkaline lysis method where QIAGEN neutralisation buffer P3 is replaced with buffer N3 and BAC DNA is precipitated from the lysate with isopropanol before being resuspended in buffer QBT then loaded onto the P20 microtip. Samples precipitated with buffer N3 perform better than buffer P3 in that they are easier to resuspend and contain less material that impedes flow of the material through the column. Following column washing and elution, DNA can be spooled out of the elution buffer by addition of isopropanol.

**Fig. 1:**
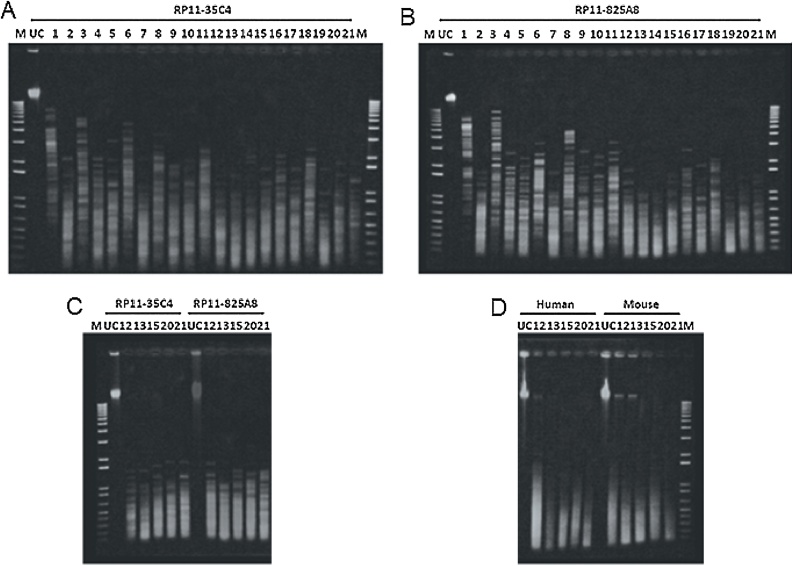
**Identification of optimal restriction enzyme combination for fragmentation.** 500 ng of BAC was digested with 5 units of restriction enzyme in the combinations outlined in Table 1 and analysed by electrophoresis on 1% agarose gel. (A) Restriction enzyme digest of BAC RP11-35C4. (B) Restriction enzyme digest of BAC RP11-825A8. Promising restriction enzyme combinations identified from images in (A) and (B) were further examined with a repeat experiment on BAC clones (C) RP11-35C4, RP11-82A8 and (D) human and mouse genomic DNA.

In order to determine the restriction enzymes suitable for fragmentation of BAC DNA to an appropriate size we selected a series of enzymes with 4bp recognition sites and the same optimal digestion buffer and tested them individually and in combination on two different BAC constructs as well as human and mouse genomic DNA (Table 1) (New England Biolabs enzymes from Genesearch, Arundel, Australia). Restriction enzymes with a 4bp recognition sequence will theoretically cut a random sequence of DNA every 4^4^ = 256bp. In practise, we found this not to be the case and to achieve fragmentation to the desired 500bp-200bp it was necessary to use a combination of two enzymes (Fig. 1). From our initial test we identified 5 enzyme combinations that exhibited promise for fragmenting DNA to the desired range of 200-500bp. These were then assessed in a side by side comparison of 6 human BAC constructs (CHORI RP11 human sequence insert BACs were sourced from MCRI, Melbourne, Australia) and human and mouse genomic DNA (Fig. 1). From a combination of the BAC and genomic DNA digests we selected the combination of CviQI and AluI as producing the best fragmentation on BAC DNA and likely to be the most consistent across all BACs.

**Table 1:** Restriction enzyme digest combination reference numbers.

Enzyme	TaqaI	AluI	MspI	HaeIII	MseI	CviQI
TaqaI	1	7	8	9	10	11
AluI		2	12	13	14	15
MspI			3	16	17	18
HaeIII				4	19	20
MseI					5	21
CviQI						6

Restriction enzyme digest combinations using NEB enzymes and NEB buffer 4 supplemented with BSA. Restriction digests performed for 1 h at 37 °C except for TaqaI (65 °C) and CviQI (25 °C). In combination reactions requiring 2 different temperatures, the lowest incubation temperature was performed first.

To evaluate whether probes made with CviQI/AluI digestion performed as well as those prepared by other methods we labelled and used them for 2 colour interphase FISH. We chose the widely utilised BACs RP11-825A8 and RP11-35C4 that flank a region in the TMPRSS2 gene that is commonly rearranged in the progression of prostate cancer Yoshimoto et al. [5]. Here we conducted FISH on the prostate cancer cell line LAPC4 that does not contain a rearrangement at the TMPRSS2 loci so the probes appear to be adjacent or overlapping. For comparative purposes we prepared probes by CviQI/AluI digestion, by sonication and, by another previously undescribed method using DNA fragmentase (New England Biolabs enzyme from Genesearch, Arundel, Australia).

Detailed methods for probe preparation are provided in the supplementary material. In brief, 1.5ug of each fragmented BAC was labelled with platinated fluorescent dyes using the universal linkage system from KREATECH (Diagnostic Technology, Belrose, Australia). RP11-825A8 was labelled 550 red/orange and RP11-35C4 was labelled with 495 green. Unincorporated fluorophore was removed using KREApure columns provided with the labelling reagent. DNA concentration and dye absorbance of the labelled material was quantitated on a Nanodrop (Thermofisher Scientific, Scoresby, Australia) and this was used to determine the degree of labelling. 1 μg of 495 green and 300 ng of 550 red/orange probes were combined with 25 ng Cot-1 DNA (Thermofisher Scientific, Scoresby, Australia) (to block non-specific binding) and this was ethanol precipitated then resuspended in 48 μl LSI/WCP hybridisation buffer (Abbott, Lane Cove, Australia). 2 μl of this was used in each hybridisation reaction. Total price of probes per FISH including DNA preparation, digest, clean up, labelling and resuspension is approximately $8.36 compared with $102.85 for commercial equivalent probes.

Standard methods for hybridisation of the probes were used (see Wiegant and Raap, [6] and Supplementary Material). Briefly, cells were harvested, hypotonically lysed, fixed in acetic acid/methanol and dropped onto slides. Slides were air dried, aged in 2XSSC for 2 min, dehydrated through an ethanol gradient and air dried. 2 μl of probe was applied to the hybridisation area and this was covered with a 13 mm round coverslip, sealed with rubber cement denatured by heating to 80 °C 15 min and left to hybridise overnight at 37 °C in a humidified chamber. The next day slides were washed in 0.4XSSC/0.3%NP40 at 73 °C for 2 min and 2XSSC/0.1% at room temperature for 1 min and allowed to air dry in the dark. Slides were then mounted in DAPI II media containing DAPI counterstain and antifade (Abbott, Lane Cove, Australia) then visualised on a microscope. Images were captured with a camera and processed with ImageJ [7].

**Fig. 2:**
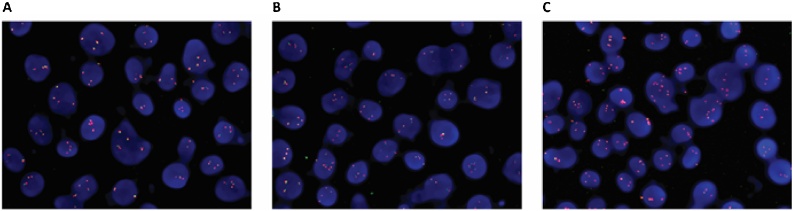
**Interphase FISH analysis with probes generated by three different fragmentation methods.** LAPC4 prostate cancer cells were subject to FISH analysis using probes prepared from non-overlapping BAC clones containing DNA sequences flanking the TMPRSS2 gene. Upstream BAC RP11-35C4 was labelled with green fluorophore and downstream BAC RP11-825A8 was labelled with red fluorophore. Nuclei were counterstained with DAPI. (A) FISH probes prepared by sonication. (B) FISH probes prepared using Fragmentase enzyme. (C) FISH probes prepared with restriction enzyme digestion using CviQ1/AluI.

FISH results obtained from probes prepared by CviQ1/AluI, Sonication and Fragmentase are comparable (Fig. 2). Use of a defined combination of restriction enzymes to prepare FISH probes provides four advantages over current methods. First it removes the requirement for extensive optimisation to produce fragments in the optimal range of 200-500bp. Second, our demonstration that this combination of enzymes produces suitable fragmentation on a number of BACs and genomic DNA indicate that it will be appropriate for fragmentation of any large insert vector and will not be influenced by sequence specific features. Thirdly, this fragmentation method relies on digest to completion rather than times attenuation of a progressive reaction and is therefore not influenced by batch specific differences in enzyme preparations. Finally it does not require access to expensive sonication equipment.

### Detailed protocol

#### Legend

 ***ATTENTION***

* ***HINT***

 ***REST***

#### Reagents

Tryptone (ThermoFisher Scientific, Scoresby, VIC, AUS)

Yeast Extract (ThermoFisher Scientific, Scoresby, VIC, AUS)

Bacto Agar (ThermoFisher Scientific, Scoresby, VIC, AUS)

Chloramphenicol (SIGMA, Castle Hill, NSW, AUS)

TRIS (SIGMA, Castle Hill, NSW, AUS)

EDTA (SIGMA, Castle Hill, NSW, AUS)

RNAse (SIGMA, Castle Hill, NSW, AUS)

SDS (SIGMA, Castle Hill, NSW, AUS)

NaOH (SIGMA, Castle Hill, NSW, AUS)

Guanidine Hydrochloride (GuHCl) (SIGMA, Castle Hill, NSW, AUS)

Potassium Acetate (KAc) (SIGMA, Castle Hill, NSW, AUS)

Potassium Chloride (SIGMA, Castle Hill, NSW, AUS)

Sodium Chloride (SIGMA, Castle Hill, NSW, AUS)

Trisodium citrate dihydrate (Na_3_Citrate ⋅ 2H_2_O) (SIGMA, Castle Hill, NSW, AUS)

NP40 (SIGMA, Castle Hill, NSW, AUS)

Acetic acid, glacial (Chem-supply, Gilman, SA, AUS)

Isopropanol (Chem-supply, Gilman, SA, AUS)

Ethanol (Chem-supply, Gilman, SA, AUS)

QIAGEN™ plasmid mini kit (QIAGEN, Chadstone, VIC, AUS)

CviQI (Genesearch, NEB, Arundel, QLD, AUS)

AluI (Genesearch, NEB, Arundel, QLD, AUS)

Phenol (SIGMA, Castle Hill, NSW, AUS)

Chloroform (SIGMA, Castle Hill, NSW, AUS)

Sodium Acetate trihydrate (NaAc) (SIGMA, Castle Hill, NSW, AUS)

Glycogen (Ambion, ThermoFisher Scientific, Scoresby, VIC, AUS, cat#9510, 5 mg/ml)

AMPure XP beads (Beckmann Coulter, Lane Cove, NSW, AUS)

KREATECH ULS™ platinum bright labelling kit (Diagnostic Technology, Belrose, NSW, AUS)

BIORAD P30 SSC spin columns (BIORAD, Gladesville, NSW)

C0t1 DNA (species specific) (Life Technologies, ThermoFisher Scientific, Scoresby, VIC, AUS, 500 μg @ 1 mg/ml Cat# 15279-011 – for human)

Hybridisation buffer Vysis LSI/WCP hybridisation buffer (Abbott, North Ryde, NSW, AUS, Cat# 06J67-001)

DAPI II counterstain (antifade/mount) (Abbott, North Ryde, NSW, AUS, Cat# 06J50-001)

#### Procedure

##### BAC preparation

1 Streak BAC stock from glycerol or stab onto LB agar with appropriate selection antibiotic (for BACPAC CHORI RP11 clones use 10 μg/ml choloramphenicol) and grow overnight at 37 °C.
2 Pick a single colony and grow in 2 ml LB + antibiotic at 37 °C during the day. Use 1 ml of turbid culture to inoculate 50 ml LB + antibiotic and grow overnight at 37 °C.
3 Pellet bacteria by centrifugation at 6000 rpm for 10 min at 4 °C.
4 Remove media and resuspend cells well in 5 ml resuspension buffer.
5 Add 5 ml lysis buffer, mix by gentle inversion of tube 7 times. Place tube on ice for 10 min.
6 Add 7 ml Guanidine−HCl neutralisation buffer, mix by gentle inversion of tube 7 times. Place on ice 10 min.
7 Pellet debris by centrifugation at 12,000 rpm for 10 min at 4 °C in a swinging bucket rotor (eg; Beckman JS13.1). Transfer supernatant to a clean tube and repeat.
8 Add 13 ml isopropanol, mix by gentle inversion of tube 7 times. Place tube on ice for 10 min.
9 Pellet BAC DNA by centrifugation at 12,000 rpm for 10 min at 4 °C in a swinging bucket rotor.
10 Gently aspirate supernatant and discard.
11 Add 750 μl 70% Ethanol to the pellet and use the liquid to wash the pellet off the bottom of the tube so that it can be transferred to a 1.5 ml tube. Perform an additional wash to obtain the remaining pellet with another 750 μl 70% Ethanol.
12 Spin down BAC DNA by centrifugation at 6000 rpm for 5 min in a microfuge.
13 Remove 70% Ethanol and air dry pellet for 10 min. Do not over dry or it will not resuspend well.
14 Add 500 μl MQ water to pellet and tube flick gently to resuspend DNA. Do not vortex.
15 Add DNA to 1.5 ml buffer QBT from QIAGEN™ plasmid mini kit.
16 Perform DNA purification on QIAGEN™ plasmid mini kit P20 columns as per manufacturer’s instructions, using the prepared BAC DNA in QBT buffer in place of the clarified bacterial cell lysate.
17 Collect DNA eluted in 800 μl EB buffer in a 1.5 ml tube. To this add 650 μl isopropanol and mix by gently inverting the tube until DNA spools out of solution.
18 Pellet the DNA with a brief spin, remove supernatant, add 500 μl 70% ethanol and mix by gently inverting the tube.
19 Spin briefly to pellet the DNA, remove supernatant and air dry DNA until just dry. Do not over dry or it will not resuspend well.
20 Resuspend DNA in 50 μl MQ water. Expected yield is >5ug total.

##### BAC fragmentation

1 Quantitate DNA and digest 3 μg with CviQI/AluI in a final volume of 50 μl in New England Biolabs buffer 4 supplemented with BSA.
2 Clean up DNA either by phenol chloroform extraction followed by ethanol precipitation (section Ci) or with AMPure para-magnetic beads (Section Cii).

##### DNA clean up with phenol chloroform

1 Make sample volume up to 100 μl with 50 μl MQ, add an equal volume (100 μl) of Phenol/chloroform and vortex briefly.
2 Centrifuge at 13,200 rpm in microfuge for 2 min.
3 Transfer upper aqueous layer to a new 1.5 ml tube, add an equal volume of chloroform and vortex briefly.
4 Centrifuge at 13,200 rpm in microfuge for 2 min.
5 Transfer upper aqueous layer to a new 1.5 ml tube.
6 Ethanol precipitate DNA (add 1/10th volume 3 M NaAc pH4.6, 0.5 μl Glycogen (5 mg/ml Ambion cat#9510) and three volumes of ethanol, incubate at −20 °C >1 h.
7 Centrifuge at 13,200 rpm in microfuge for 10 min. Remove supernatant.
8 Wash pellet by adding 500 μl 70% EtOH, mix by gentle inversion
9 Centrifuge at 13,200 rpm in microfuge for 5 min and let pellet air dry.
10 Resuspend pellet in 10 μl MQ water.

##### DNA clean up with ampure pARA-mAGNETIC beads

1 Allow AMPure XP beads to equilibrate to room temperature and vortex to resuspend.
2 Add 45 μl (0.9X) beads to restriction digest, vortex briefly and leave at room temperature for 5 min.
3 Place tube in magnetic rack.
4 When solution clarifies and beads migrate to the magnet, remove and discard supernatant.
5 Wash beads by adding 200 μl 80% ethanol to tube.
6 After 30 s remove 80% ethanol and repeat wash.
7 After second wash remove all visible liquid and allow to air dry for 5 min
8 Resuspend pellet in 10 μl MQ water.

##### Fish probe labelling and clean up

1 Quantitate fragmented DNA on a NanoDrop™ and prepare 1 μg DNA in 16 μl MQ.
2 From the KREATECH ULS Platinum Bright™ nucleic acid labelling kit add 2 μl 10X labelling mix and 2 μl ULS label.
3 Incubate at 85 °C for 30–45 min.
4 Equilibrate to room temperature.
5 Remove unincorporated label using columns and protocol provided in labelling kit. It is also possible to use BIORAD P30 SSC spin columns but the reaction volume should be made up to 50 μl with MQ prior to applying labelled probe to column. If using BIORAD P30 SSC spin columns keep some of the column equilibration buffer to zero the NanoDrop™ in the next step.
6 Use NanoDrop™ set on “labelling” to read absorbance at fluorophore λ_max_ (Table 2) and A_260_ (path length = 1 cm)

**Table 2:** Spectral properties of the KREATECH fluorophores, including correction factors.

KREATECH Catalogue #	Product Name	ULS dye	λ_max_ (nm)	Em (nm)	EC (e max)	CF (CF_260_)
GLK-001	Platinum Bright 495 Green	Fluorescein	495	517	83,000	0.32
GLK-002	Platinum Bright 547 Red/Orange	Dyomics547	547	565	1,50,000	0.08
GLK-003	Platinum Bright 647 Infrared	Dyomics647	647	665	2,50,000	0.05
GLK-004	Platinum Bright 550 Red/Orange	Rhodamine	550	573	91,000	0.33
GLK-005	Platinum Bright 570 Red/Orange	d-Red	570	591	1,29,000	0.3
GLK-006	Platinum Bright 415 Blue	Dyomics415	415	472	56,000	0.3

λ_max_; absorbance maximum. Em; emission maximum. EC; extinction coefficient at λ_max_ (cm^−1^M^−1^). CF260; correction factor at A_260_.

##### Calculating probe concentration and degree of labelling

1 To correct for the influence of fluorophore on the A_260_ reading use the following formulae and correction factors provided in Table 1.

A_base_ = A_260_ – (A_dye_ – CF_260_)

[Probe ng/μl] = A_base_ x 50

2 To calculate the degree of labelling (DOL), or ratio of bases to dye molecules use the following formula (ε_base_ (cm^−1^M^−1^) for dsDNA = 6600).

DOL = (A_base_ x ε_dye_)/(A_dye_ x ε_base_)

3 A degree of labelling <60 (ie: more than 1 labelling molecule per 60bp) will give an acceptable signal in FISH.

##### Preparation of probe for fish

1 Add together; 1 μg of green probe, 300 ng red probe, 25 μg C0t-1 DNA in a microfuge tube, note total volume and ethanol precipitate nucleic acids (add 1/10th volume 3 M NaAc pH4.6 and three volumes of ethanol, mix and incubate at −20 °C >1 h.)
2 Centrifuge at 13,200 rpm in microfuge for 10 min. Remove supernatant.
3 Wash pellet by adding 500 μl 70% EtOH, mix by gentle inversion.
4 Centrifuge at 13,200 rpm in microfuge for 5 min and let pellet air dry.
5 Resuspend in 48 μl hybridisation buffer (Abbott Vysis LSI/WCP hybridisation buffer).
6 Use immediately or store at −20 °C.

##### Mounting cells on slides

1 The following protocol has been optimised for cells derived from one well of a 6 well plate (2 × 10^5^ – 8 × 10^5^ cells).
2 To prepare slides for use store in ethanol, rinse with RO water, dry, wrap in a clean Kimwipe and store at 4 °C.
3 Pre-warm 0.075 M KCl to 37 °C water bath.
4 Lift cells as per usual practice (eg: Trypsin/EDTA).
5 Once cells have lifted, resuspend in media and transfer to labelled centrifuge tube.
6 Centrifuge the specimen for 4 min at 1300 rpm.
7 Remove the supernatant and resuspend the cell pellet in 8 ml of pre-warmed KCl.
8 Incubate in a 37 °C water bath for 15 min.
9 Add 2 ml of 3:1 methanol:glacial acetic acid fixative to the hypotonic solution and mix well by pipetting or inversion.
10 Centrifuge the suspension for 4 min at 1300 rpm.
11 Remove the supernatant and resuspend the pellet in 4–5 ml 3:1 methanol:glacial acetic acid fixative.
12 Centrifuge the suspension for 4 min at 1300 rpm.
13 Remove most of the supernatant, leaving only a small volume of liquid (40–60 μl) to resuspend the pellet.

* ***HINT***

Fixed cells can be stored for up to 3 months at −20 °C after resuspension in 2 ml 3:1 methanol:glacial acetic acid fixative.

14 Drop 10 μl of cell suspension from a height of 10 cm onto cold, dry slides held at an angle (approximately 30° relative to the length of slide). Use a diamond pencil to mark the location of cells and label the slides (label must give slide a clear cells-up-down orientation). Check cell density and if not adequate, more suspension may be added to the area.
15 Allow drop to air dry.

* ***HINT***

Add 2 ml fixative to the remaining cells in the tube and store at −20 °C until sample is no longer required (max 3months).

##### Probe hybridisation

1 When slides are air dry, let them “age” by placing in 2X SSC for 2 min.
2 Dehydrate slides for 2 min each through 70%, 80% and 100% ethanol and air dry at room temperature.
3 Place slides on a 37 °C surface and apply 2 μl probe to each hybridisation area. Cover with a 13 mm round coverslip and seal with rubber cement.
4 Place slides on a flat surface in 85 °C oven for 20 min.
5 Remove slides from oven and place in 37 °C humidified chamber overnight.

##### Post hybridisation wash and mount

1 Pre-warm a Coplin jar containing 0.4XSSC/0.3%NP40 in a 73 °C ( ± 1 °C) water bath.
2 Prepare another Coplin jar with 2XSSC/0.1%NP40 at room temperature
3 Take the slide from the humidified chamber, remove the rubber cement with a needle and flick off the coverslip
4 Immediately place the slide into the preheated 0.4XSSC/0.3% NP40 (73 °C) water bath.
5 Agitate for approximately 3 s and then incubate for 3min

* ***HINT***

Do not place more than 4 slides in the wash at one time. Begin timing when the last slide has been added to the wash.

6 Remove the slide and place in the 2XSSC/0.1% NP40 at room temperature agitate for 1–3 sec and then incubate for 30–60 sec.
7 Remove the slide, wipe off excess liquid with a tissue taking care not to wipe area with cells and allow to air dry in the dark (eg: in a closed drawer)
8 Apply 10 μl of the DAPI counterstain/antifade mount (Abbott) each hybridisation area and cover with an 18mm^2^ square coverslip.
9 Seal edges with clear nail polish and allow it to dry in the dark.
10 Examine slides immediately or protect from light and store in at −20 °C.

##### Recipes

**Table:** 

1 LB - Luria Broth (1l)	
Ingredient	Amount
Tryptone	10g
Yeast extract	5g
NaCl	10g

**Table:** 

2 LB agar (1l)	
Ingredient	Amount
LB	1l
Bacto Agar	15g

**Table:** 

3 Chloramphenicol (10ml)		
Ingredient	Amount	Final Concentration
Chloramphenicol	100mg	10mg/ml
Ethanol	10ml

**Table:** 

4 RNAse (10ml)		
Ingredient	Amount	Final Concentration
RNAseA	100mg	10mg/ml
MQ water	10ml	
SIGMA 4875-100MG RNAseA does not contain DNAse so it is not necessary to boil stock.

**Table:** 

5 Resuspension buffer (100ml)			
Ingredient	Stock	Amount	Final Concentration
TRIS-HCl pH8.0	1M	5ml	50mM
EDTA	0.5M	2ml	10mM
RNAseA	10mg/ml	1ml	100μg/ml

**Table:** 

6 Lysis Buffer (100ml)			
Ingredient	Stock	Amount	Final Concentration
NaOH	10M	2ml	200mM
SDS	10% (w/v)	10ml	1% (w/v)

**Table:** 

7 Guanidine-HCL neutralisation buffer (250ml)		
Ingredient	Amount	Final Concentration
GuHCl	100.3g	4.2M
KAc	22.1g	0.9M
Adjust to pH4.8 with glacial acetic acid (approx. 10ml)		

 ***ATTENTION***

8 Phenol is highly caustic, use appropriate handling procedures
9 Melt solid phenol by incubating at 65 °C
10 Add an equal volume of chloroform and mix by swirling
11 To saturate the phenol and alter the pH, add some 0.1 M TRIS−HCl pH8.0 and mix
12 Allow the mix to partition into aqueous (top) and organic (bottom) phases
13 Remove the aqueous phase and repeat equilibration 2 more times
14 For short term use, store at 4 °C or freeze stocks
15 Do not use oxidised (dark pink) solutions

**Table:** 

9 Sodium Acetate pH5.2 (100ml)		
Ingredient	Amount	Final Concentration
NaAc ⋅ 3H_2_O	4.563g	3M
Adjust to pH5.2 with glacial acetic acid

**Table:** 

10 Methanol:Glacial Acetic Acid fixative (3:1)		
Ingredient	Amount	Final Concentration
Methanol	37.5ml	75% (v/v)
Acetic Acid	12.5ml	25% (v/v)

**Table:** 

11 20XSSC (1l)		
Ingredient	Amount	Final Concentration
NaCl	175.32g	3M
Na_3_Citrate ⋅ 2H_2_O	88.23g	0.3M
Adjust to pH7.0 with HCl		

##### Equipment

Beckman J series centrifuge (Beckmann Coulter, Lane Cove, NSW, AUS)

Beckman JS13.1 Swinging bucket rotor (Beckmann Coulter, Lane Cove, NSW, AUS)

Eppendorf refrigerated microcentrifuge (Eppendorf, North Ryde, NSW, AUS)

NanoDrop (ThermoFisher Scientific, Scoresby, VIC, AUS)

Kimwipe (ThermoFisher Scientific, Scoresby, VIC, AUS)

Waterbath (RATEK, Boronia, VIC, AUS)

Coplin Jar (ThermoFisher Scientific, Scoresby, VIC, AUS)
